# Optimizing the thermostability of triketone dioxygenase for engineering tolerance to mesotrione herbicide in soybean and cotton

**DOI:** 10.3389/fpls.2024.1502454

**Published:** 2025-02-04

**Authors:** Stephen M. G. Duff, Lei Shi, Danqi Chen, Xiaoran Fu, Mingsheng Peng, Clayton T. Larue, Janice Weihe, Jessica Koczan, Brian Krebel, Qungang Qi

**Affiliations:** ^1^ Bayer Crop Science, St. Louis, MO, United States; ^2^ Bayer Crop Sciences, Chesterfield, MO, United States; ^3^ Encodia Inc., San Diego, CA, United States; ^4^ Ionova Life Science, Shenzhen, China; ^5^ Bayer Crop Science, Diegem, Belgium

**Keywords:** protein engineering, triketone dioxygenase, 4-hydroxyphenylpyruvate dioxygenase (HPPD) inhibitors, protein variants, weed management

## Abstract

Optimized triketone dioxygenase (TDO) variants with enhanced temperature stability parameters were engineered to enable robust triketone tolerance in transgenic cotton and soybean crops. This herbicide tolerance trait, which can metabolize triketone herbicides such as mesotrione and tembotrione, could be useful for weed management systems and provide additional tools for farmers to control weeds. TDO has a low melting point (~39°C–40°C). We designed an optimization scheme using a hypothesis-based rational design to improve the temperature stability of TDO. Temperature stabilization resulted in enzymes with K_cat_ values less than half of wild-type TDO. The best variant TDO had a K_cat_ of 1.2 min^−1^ compared to wild-type TDO, which had a K_cat_ of 2.7 min^−1^. However K_m_ values did not change much due to temperature stabilization. Recovery of the K_cat_ without losing heat stability was the focus of additional optimization. Multiple variants were found that had better heat stability *in vitro* and efficacies against mesotrione equaling the wild-type (WT) TDO in greenhouse and field tests.

## 1 Introduction

Improving plant productivity in a sustainable manner has become increasingly important to meet the food and energy requirements of an expanding global population. Weed control is important for managing sustainable crop production, as weeds compete with crops for water, nutrients, sunlight, and physical space (Popp et al., 2013). Synthetic herbicides are an important tool to control weeds (Green, 2012). However, broad-spectrum herbicides may injure crops. Genetic modification of crops, which imparts tolerance to herbicide application, is an important tool, enabling farmers to selectively control weeds (Goyal and Lichtfouse, 2015).

Herbicide use has resulted in the movement of weed populations, through selection, to heritable traits conferring phenotypic resistance (i.e., mechanisms protecting plants by a reduction in herbicide damage, which allows weeds to flourish despite the herbicide application; Busi et al., 2013; Nandula, 2019).

Herbicide resistance increases the cost of weed management and limits the herbicide options to control resistant weeds (Peterson et al., 2018). Developing and deploying crops with tolerance to diverse herbicide sites of action as trait stacks is essential to enable farmers to effectively manage weeds. Crops with stacked herbicide tolerance traits will also help slow the pace of herbicide-resistant weed evolution (Beckie et al., 2019; Larue et al., 2019, 2020; Nandula et al., 2019).

Tyrosine catabolism has a vital role in plant metabolism due to the synthesis of plastoquinone. Plastoquinone is a downstream product in the tyrosine catabolism pathway and is required for photosystem II function and carotenoid synthesis (Kraehmer et al., 2014; Lichtfouse, 2018). 4-Hydroxyphenylpyruvate dioxygenase (HPPD; EC: 1.13.11.27) catalyzes the first committed step of tyrosine catabolism in a reaction that involves decarboxylation, aromatic oxygenation, and substituent migration. HPPD incorporates two atoms of molecular oxygen to form homogentisate from 4-hydroxyphenylpyruvate (Lindblad et al., 1970).

HPPD inhibitors are among the most recently discovered herbicide sites of action (Norris et al., 1998; van Almsick, 2009). One potent chemical class of HPPD inhibitors is the triketones, which include mesotrione (Mitchell et al., 2001). They are widely applied as herbicides for use in monocot crops such as maize to control dicot weeds (Beaudegnies et al., 2009).

Maeda et al. recently identified the rice HPPD INHIBITOR SENSITIVE 1 (Triketone dioxygenase, TDO) gene, which is responsible for the tolerance to benzobicyclon and mesotrione, based on varietal variation in sensitivity to this herbicide (Maeda et al., 2019). We recently overexpressed TDO in soybean, imparting resistance to mesotrione and other triketones (Dai et al., 2022).

Current advances in rational design, computation, and directed evolution along with parallel advances in plant breeding and omics technologies offer enormous opportunities for genetic engineering novel traits into the next generation of crops (Rao, 2008). Recently, we successfully engineered a microbial-sourced dioxygenase to improve enzymatic parameters, including enhanced enzymatic activity of an α-ketoglutarate-dependent (*R*)-dichlorprop dioxygenase for use with selected herbicides and improved temperature stability (Larue et al., 2019).

Surprisingly, we found that TDO, a native rice protein, had a low *in vitro* melting point of 39.5°C (**Table 1**). Since proteins begin to melt several degrees Celsius below their half-way melting point, there is a potential for low-temperature stability to reduce or eliminate the protection of TDO against triketones if the elevated temperature in the field coincides with herbicide application. Therefore, we were interested in applying modern enzyme optimization techniques to TDO to improve temperature stability. A more temperature-stable TDO could provide a backup or be useful for crops growing in higher-temperature environments or regions. Experience from this optimization effort could be applied to other biotech traits in which enzymes may be optimized to function under a range of environmental conditions.

**Table 1:** Kinetic analysis, thermostability, and R0 soybean plant injury ratings of TDO round 1 variants.

Protein	K_cat_ (min^−1^)	K_m_ (mesotrione) (μM)	K_m_ (α-ketoglutarate) (mM)	K_d_ (Fe(II)) (μM)	K_cat_/K_m_ [M^−1^ sec^−1^] at 25°C (mesotrione)	T_m_ (°C)	40°C half-life (apoprotein) (min)	Half-life of mesotrione, 200 µM Fe(II), 1 mM α-ketoglutarate (min)	R0 injury rate (no. plants/total plants with less than 3% injury	Expression (ppm)
Os.TDO	2.70 ± 0.10	6 ± 1	0.11 ± 0.03	7.1 ± 2.1	7,528	39.5	4.5 ± 0.4	30.7 ± 3.5	1.67 (3/3)	N/A
D08	1.20 ± 0.08	20 ± 4	0.12 ± 0.03	8.8 ± 2.8	967	46	49.6 ± 0.3.2	117.9 ± 23.3	3.33 (6/7)	9.42
B03	1.20 ± 0.07	8 ± 2	0.09 ± 0.03	2.5 ± 0.9	2,167	51	>120	125.9 ± 32.5	6.43 (7/7)	13.88
F09	0.70 ± 0.03	38 ± 4	0.14 ± 0.04	2.8 ± 0.9	316	53.5	>120	94.2 ± 21.9	11.43 (7/7)	8.61
A04	0.40 ± 0.04	45 ± 11	0.13 ± 0.05	4.2 ± 1.6	138	46	18.3 ± 5.1	nd	24.29 (5/7)	25.55
B09	0.13 ± 0.03	118 ± 49	0.10 ± 0.05	0.4 ± 0.5	18	49	8.8 ± 3.4	nd	16.43 (7/7)	16.43

1 mM ketoglutarate and 200 µM Fe were added where indicated. Kinetic parameters are those of the second hydroxylation unless otherwise indicated. Expression was determined by ELISA.

TDO, triketone dioxygenase.

## 2 Materials and methods

### 2.1 Cloning, expression, and purification of TDO variants

TDO and its variants were cloned, expressed, and purified as previously described for FT_T (Larue et al., 2019).

### 2.2 Transformation of soybean and cotton with TDO constructs

Embryos from soybean (AG3555 germplasm) were transformed using *Agrobacterium*-mediated transformation with constitutive plant expression vectors driving a TDO and its variants as previously described (Dai et al., 2022; Larue et al., 2020). Similarly, transgenic cotton plants were generated via *Agrobacterium*-mediated transformation following the protocol outlined by Chen et al. (2014) using DP393 as the transformation germplasm. TDO copy number was determined via a TaqMan assay. Transgenic soybean or cotton events carrying a single-copy TDO insertion were used for herbicide tolerance and thermostability evaluations in this study. Control soybean and cotton plants (wild type) are plants that were not transformed with TDO or its variants but otherwise treated and advanced in the same nurseries and the same manner as the transgenic plants (cotton germplasm DP393).

### 2.3 TDO kinetic and thermostability assays

The activity and kinetics of the first and second hydroxylations of mesotrione catalyzed by TDO and its variants were assayed as previously described (Duff et al., 2024). The Michaelis–Menten plots were calculated using at least eight separate concentrations of substrate. Three different assays were used to assess the temperature dependence and stability of TDO, including T_m_ and k_cat_/k_m_ at 25°C, 30°C, 35°C, and 40°C, and the half-life of active TDO (residual activity and K_cat_/K_m_) at 40°C (Duff et al., 2024). To measure the thermostability half-life of TDO, 2 μg protein was added to a 96-well plate and incubated for 0 min, 5 min, 10 min, 15 min, 20 min, 30 min, 60 min, and 90 min. The plate was cooled to room temperature, and then the resulting reaction mixture was supplemented with the missing substrates and cofactors to initiate the TDO reaction. The residual activity of heat-treated TDO was measured by activity. All reaction rates were normalized to the zero timepoint.

These *in vitro* assays are intended to predict the performance and resistance to mesotrione of plants expressing variants of TDO, including the amount of residual enzyme activity that would be expected after prolonged heat exposure (e.g., night-time tolerance *in vivo*), and plant performance at the onset of elevated temperature conditions.

### 2.4 Engineering variants for temperature stability

**Figure 1:**
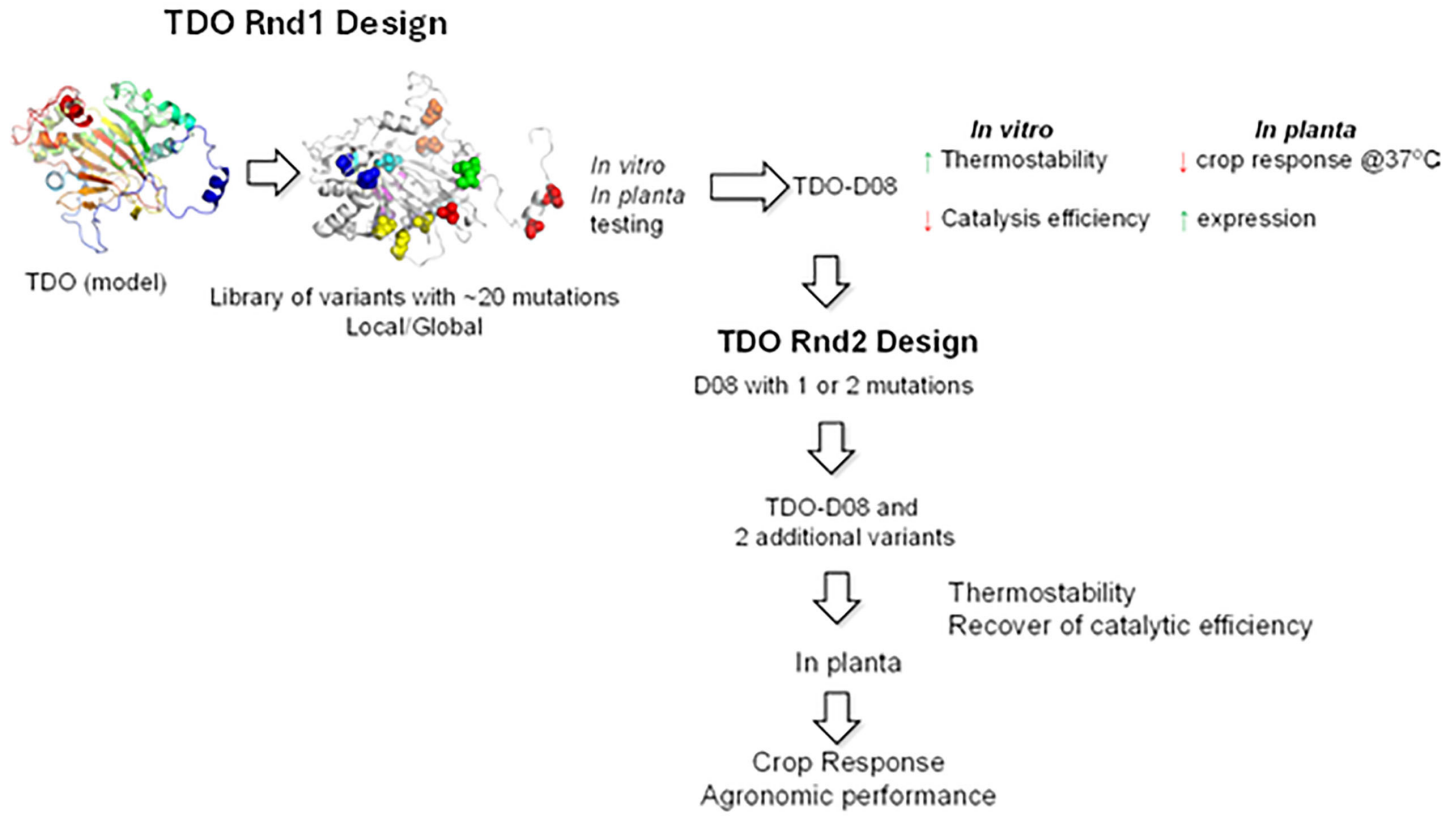
General optimization scheme for TDO. There were two rounds of optimization. The first round focused on improving thermostability. The second round focused on restoring the catalytic activity lost in the first round. Enzymes were evaluated using several measurements of thermostability and kinetics. Plants were evaluated in a controlled environment for mesotrione tolerance and in the field for mesotrione tolerance and agronomic performance. TDO, triketone dioxygenase.

The general strategy for optimizing the temperature stability of TDO (**Figure 1**) was deployed over two rounds of engineering. In the first round, an attempt was made to improve the temperature stability of TDO. However, this resulted in a general loss of activity and poorer kinetic parameters. Therefore, a second round was undertaken to restore enzyme activity and kinetics while maintaining thermostability. Within each round of engineering, multiple sequential assays were employed to screen variants for advancement. For each round of engineering, the final stages of testing for those few variants that made it through the earlier stages were focused on.

In round 1, an attempt was made to improve the thermostability of TDO. TDO sequence information through mining and a TDO model were combined to select the positions and residues for mutation. A total of 82 out of 351 positions were selected to mutate to the most conserved residues using Multiple Sequence Alignment (MSA) analysis. A total of 20 mutations were made on the protein by combining the most probably single mutations identified from the previous step. Two different combination strategies were designed: 1) combine mutations that spread out throughout the protein and 2) combine mutations that are clustered together. A library of variants was then created with 10 local and 10 global mutations such that each variant had a total of 20 mutations (**Figure 1**). Variants were screened *in vitro* (thermostability and catalytic efficiency) and *in planta* (injury rate at elevated temperature and protein expression). Variants were initially screened and advanced based on their thermo melting point (T_m_) and specific activity at two concentrations of mesotrione. The kinetic parameters of the best variants were determined at 25°C and elevated temperatures. In the final stage of round 1, the half-life of the best TDO variants was determined. The best variants were then introduced into soybean and tested in the greenhouse at normal and elevated temperatures. The final criteria for picking the round 1 winner were *in vitro* thermostability, *in vitro* catalysis efficiency, plant TDO protein expression, and plant injury when treated with mesotrione at 37°C.

In round 1, one variant (D08) was chosen, which had the best thermostability, while retaining >50% of the activity (**Figure 1**). Most of the rational design work in round 2 focused on selected point amino acid changes in D08 (relative to wild-type) that were made in round 1, but those in round 2 were restored to wild-type amino acids (or similar amino acids to wild type) with the general approach being that the amino acid positions could be uncovered that were important for activity and those that were important for stability. The goal was to find amino acid positions that were independent of each other so that improvements could be made to both stability and activity without a negative impact on the opposing improvement target to build variants with improved characteristics for both objectives. More simply, the focus was on separating the thermostability and kinetic traits of TDO.

We set up a design and testing strategy to improve the catalytic efficiency of thermal stable D08. The hypothesis is that during the process of making a thermal variant, some of the mutations have impacted the catalytic site even though we took extra care to avoid those sites based on a homology model. The design strategy is to systematically revert mutations from D08 to wild-type (WT) residues. There are 20 mutations in D08 relative to WT, and we can cover all single and double mutation reversions. Since it is not practical to cover the reversion of three residues out of 20 mutations, we clustered 20 mutations based on their spatial arrangement, resulting in seven distinct clusters. We then reverted groups back to WT. Since there were only a small number of clusters, we also have designs that go from WT to D08 by adding each cluster systematically.

Another hypothesis is that enzyme activity is decreased with increased rigidity or thermal stability. In this case, reversion to WT will not be able to gain back catalytic efficiency while maintaining thermal stability. We made additional designs on the putative binding site in the D08 backbone based on homology. Completely conserved residues were mutated to alanine or glycine. Those mutations will serve as internal negative controls in the experiments.

In summary, there were 324 round 2 TDO designs that have four different categories:

1. Reversion of D08 mutations to TDO-WT (both single 20 and double 190),
2. Group of mutations on D08 (total of 7) based on the spatial arrangement and revert groups back to WT (both single and combinations, total of 29),
3. Like (2), except adding the groups back to WT (2), and
4. Mutations on the putative binding site of mesotrione in the D08 backbone. Totally conserved residues (12) were mutated to A or G (as a negative control, also help study mode of action (MOA)). Semi-conserved residues were sampled according to homology scan (45).

Improving an enzyme for either stability or activity often has an opposing effect on the other characteristic. For example, improving stability may result in an enzyme that is very resistant to denaturing at higher temperatures, but at the expense of reducing overall enzymatic activity. Effort was focused on improvements in both characteristics at the same time, which is often a much larger engineering challenge. The library of 270 round 2 variants also included one or two additional mutations other than D08 based on specific hypotheses. Sequential steps of screening were undertaken to identify the best variant, including estimation of V_max_, k_cat_/k_m_, and k_cat_/k_m_ at 25°C, 30°C, 35°C, and 40°C, and half-life of active TDO at 40°C. Finally, the best variants (with T_m_ of 45°C or better and K_cat_ greater than 50% of the WT TDO) were introduced into cotton and tested for efficacy in cotton and soybean in a controlled environment and finally field trials. More specifically, in rounds 1 and 2, a small number of the best variants were advanced, which could be reasonably assayed both *in vitro* and *in planta*.

### 2.5 Controlled environment temperature evaluation of TDO variants in soybean and cotton

For soybean, 22 transgenic soybean events (16 plants per event) from five constructs expressing wild-type and four round 1 variants of TDO (**Table 1**) under a 35S promoter were grown in a greenhouse (GH) with an average daytime temperature of approximately 27°C and night temperature at 23°C. At the V3 developmental stage, half of the transgenic plants for each event were moved to a growth chamber (37.2°C day; 27.8°C night; 60% relative humidity), while the other half was continuously grown in the greenhouse. Three days after moving the plants to the growth chamber, leaf samples were collected from the top-developed leaf of each plant in the growth chamber and greenhouse and tested by ELISA (detection limit = 0.6 parts per million) to determine TDO protein expression. Subsequently, mesotrione at 420 g/ha (4×) was sprayed on all the plants in the experiment. Eight wild-type plants were sprayed with 4× mesotrione as a non-transgenic control. Herbicide treatments under greenhouse conditions were applied using a track-mounted sprayer in an enclosed cabinet calibrated to deliver 15 gallons per acre (GPA) using Teejet^®^ Air Induction (TTI) nozzles with water as the herbicide carrier. Visual injury ratings were taken at 7–14 days after mesotrione application on a scale of 0% to 100%, with “0%” being no injury and “100%” being plant death, relative to the untreated transgenic and wild-type controls. At 12 days after mesotrione spray, three plants from each event were sampled and evaluated using ELISA for leaf protein expression.

Similarly, for cotton, 12 transgenic cotton events (32 plants per event) from four constructs expressing wild-type TDO, D08, and two round 2 variants under a constitutive promoter (CUCme.Cab1) were grown in a GH with temperatures of approximately 27°C day/21°C night. At the V3–V4 developmental stage, half of the transgenic plants for each event were moved to a hot growth chamber (37.2°C at both day and night), while the other half was continuously grown in the GH. Three days after moving the plants to the hot growth chamber (GC), mesotrione at 210 g/ha and 840 g/ha was sprayed on all the plants in both GH and GC. Sixteen wild-type cotton plants were sprayed with mesotrione as a non-transgenic control. At 12 days after mesotrione spray, leaf samples from four plants for each event were collected and evaluated using ELISA for protein expression (detection limit for cotton = 1.25 parts per million). Visual injury ratings were taken 12 days after the mesotrione application.

TDO 1.2 min^−1^

### 2.6 Crop field trials

Field trials for soybean and cotton were performed as previously described (Larue et al., 2020). Both soybean plants expressing the TDO D08 variant (pMON368779) or wild-type TDO constructs (pMON290249) were tested in four 2017 US Illinois field sites with three replications per site in a randomized complete block design. In the efficacy trials, mesotrione at 210 g ai/ha was applied at the V3 growth stage followed by the V8 stage using a tractor-mounted or backpack sprayer calibrated to deliver 15 GPA with water as the herbicide carrier using TTI nozzles. Visual injury ratings were taken 7–14 days after treatments on a scale of 0 to 100, with “0” being no visible crop injury and “100” being complete crop injury or death. A similar protocol was followed in cotton field trials. Cotton plants expressing TDO D08 variant (pMON415301) or wild-type TDO constructs (pMON324616) were grown in 2019 field trials at eight locations with three replications across the cotton belt. Mesotrione treatments at 210 g ai/ha were applied at the pre-emergent and V4 stages followed by the early bloom (V8) stage. Visual crop injury ratings were collected 7–14 days after the treatment. All data from field trials were subjected to analysis of variance and means separated by least significant difference (LSD) at alpha 0.05. Statistical analysis was automated using the ASReml software for mixed model fitting.

### 2.7 Tissue collection and extraction for ELISA quantitation

Soy or cotton leaf tissues expressing Os.TDO from different treatment groups were collected on dry ice at indicated growth stages in a greenhouse. The tissues were lyophilized, weighed before extraction in detergent-containing ELISA buffer at 1:100 of tissue:buffer ratio, and then ground with three chrome steel beads for 2 min at 1,000 RPM on a mega grinder. The crude extract was centrifuged at 1,865 × *g* for 10 min at 10°C. An equal volume of supernatant was transferred into 1-mL matrix tubes and stored at −8°C until analysis by ELISA.

Two antibody sandwich ELISA was used for Os.TDO protein quantitation. The specific capture antibody was coated to the 384-well plate at 5 μg/mL overnight at 4°C. The next day, the extra antibody was washed off. Purified Os.TDO protein standard and diluted samples were added to the plate and incubated for 1 hr at 37°C. The plate was washed again to remove non-bound materials and then added with a biotin-labeled detection antibody. After 1-hr incubation at 37°C, the plate was washed, added with streptavidin–horseradish peroxidase (HRP) conjugate, and underwent incubation for 1 hr at 37°C again. The signal in the plate was developed with HRP substrate 3,3′,5,5′-tetramethylbenzidine (TMB; Sigma-Aldrich, St. Louis, MO, USA; #T0440) for 10 min before being stopped with 3 M sulfuric acid. The plate was read in a microplate reader (Molecular Device, San Jose, CA, USA; SpectraMax 384 PLUS) and analyzed using the SoftMax Pro 5.0 software. The final protein concentration in leaf tissues was calculated with the final dilution of extracted samples in ELISA and reported as PPM against the dry weight of leaf tissues. The detection sensitivity of Os.TDO ELISA is 0.63 ppm.

## 3 Results

### 3.1 Rationale for improving TDO thermostability

**Figure 2:**
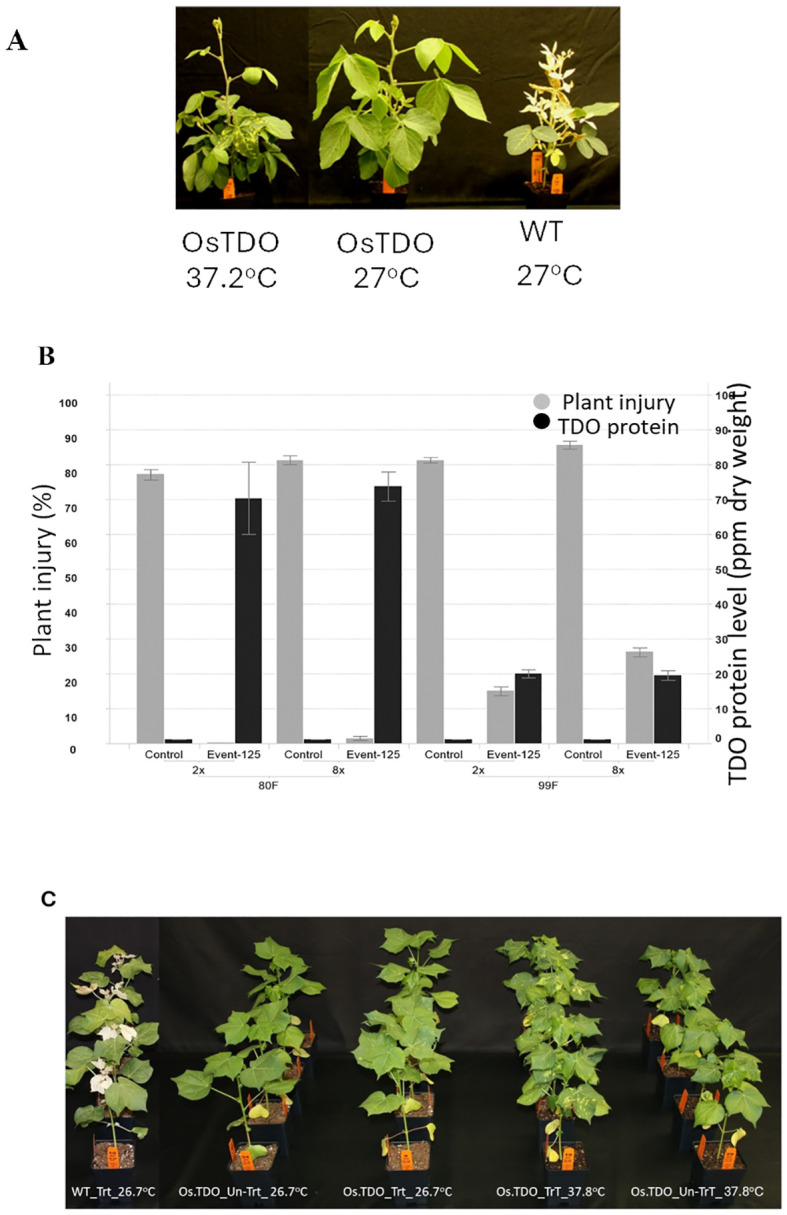
Herbicide injury and TDO expression level in WT and TDO soybean plants. **(A)** Os.TDO-transgenic soybean event 125 treated with mesotrione grown at 37.2°C (left) or 27.0°C (middle) and treated wild-type plant grown at 27.0°C (right). **(B)** Correlation of increased mesotrione injury to decreased TDO protein levels in transgenic soybean plants grown under normal (27°C, left) and elevated (37.2°C, right) temperature conditions. The black bars represent TDO protein levels in parts per million per dry weight; the gray bars represent plant injury rate (%) to 2× and 8× mesotrione treatments. **(C)** Os.TDO wild type and two transgenic cotton events grown at 37.2°C untreated or treated with 1×, 2×, or 8× mesotrione. **(C)** Os.TDO-transgenic cotton plant response to high temperature at 37.2°C. WT, wild-type plant; Os.TDO, wild-type Os.TDO-transgenic cotton plants; UTC, untreated with mesotrione; TrT, treated with mesotrione at 840 g/ha.

**Figure 2** shows the effect of elevated temperature on mesotrione injury and its correlation to decreased TDO protein level as detected by ELISA in growth chamber-grown soybean and cotton plants. Os.TDO-transgenic soybean plants and wild-type control plants were grown at 27.0°C (daytime temperature) until the V3 growth stage, and then half of the plants were transferred to 37.2°C (daytime temperature. After 3 days, leaf samples were harvested to determine TDO protein levels, and plants were then treated with 2× and 8× rates of mesotrione. Crop injury ratings were taken 11 days after mesotrione treatment. WT-TDO soybean showed no injury at 27.0°C, but some injury was apparent at 37.2°C. TDO levels in the transgenic plants were much lower at 37.2°C, suggesting that TDO may be less stable at the higher temperature. Similar effects with a lower degree of high temperature were observed in Os.TDO cotton plants (**Figure 2C**; **Table 2**). This lower temperature stability may have been responsible for the moderate injury that the plants experienced at 37.2°C, although the TDO-expressing plants also used proof of concept expression elements that had not been optimized.

**Table 2:** Cotton injury and TDO protein expression in plants expressing TDO round 2 variants at two temperatures and mesotrione treatments.

Construct	TDO variant	Crop Injury				TDO protein expression			
		% averaged crop injury^1^, under 26.7°C temperature		% averaged crop injury^1^, under 37.8°C temperature		^2^TDO levels, ppm dry weight, under 26.7°C temperature		TDO levels, ppm dry weight, under 37.8°C treatment for 12 days	
		210 g/ha mesotrione	840 g/ha mesotrione	Untreated	840 g/ha mesotrione	Untreated	210 g/ha mesotrione, 9 DAT	Untreated	210 g/ha mesotrione, 9 DAT
None	Wild-type	50	52.5	63.8	71.9	nd	nd	nd	nd
pMON415301	D08	0	0	0	0	67.5 ± 17	65.1 ± 19.3	49.6 ± 15.2	57.7 ± 7
pMON415986	Round 2—205	0	0	0	0	57.3 ± 17.8	51 ± 13.8	54.2 ± 15.9	50.5 ± 9.9
pMON415988	Round 2—225	0.25	0	0.27	0	55.5 ± 22	53.3 ± 13.9	48.9 ± 13.3	55.2 ± 15.9
pMON324616	Os.TDO	0	6.6	14.3	23.1	50.9 ± 13.7	41.5 ± 8.8	27.5 ± 3.8	24.9 ± 5

DAT, days after mesotrione treatment; nd, not detectable (0.75 ppm); TDO, triketone dioxygenase.

^1^Averaged across 16 R2 plants treated with and without 2× mesotrione.

^2^Averaged from four replicates of V4 leaf samples (4 R2 plants).

### 3.2 Kinetics and thermostability of round 1 TDO variants

The library of round 1 variants was screened as described in the Materials and Methods. The *in vitro* kinetic and thermo parameters and soybean R0 injury rate for the five best round 1 variants are shown in **Table 1**. All variants had lower K_cat_, higher K_m_, and lower K_cat_/K_m_ values (specificity constants) relative to wild-type TDO. However, all the variants had higher T_m_ and 40°C activity stability than wild-type TDO. In addition, the K_cat_/K_m_ of the variants at elevated temperatures was closer to that of wild-type TDO compared to 25°C (data not shown).

### 3.3 The effect of temperature on the protein accumulation and injury rate of round 1 variants in soybean

**Figure 3:**
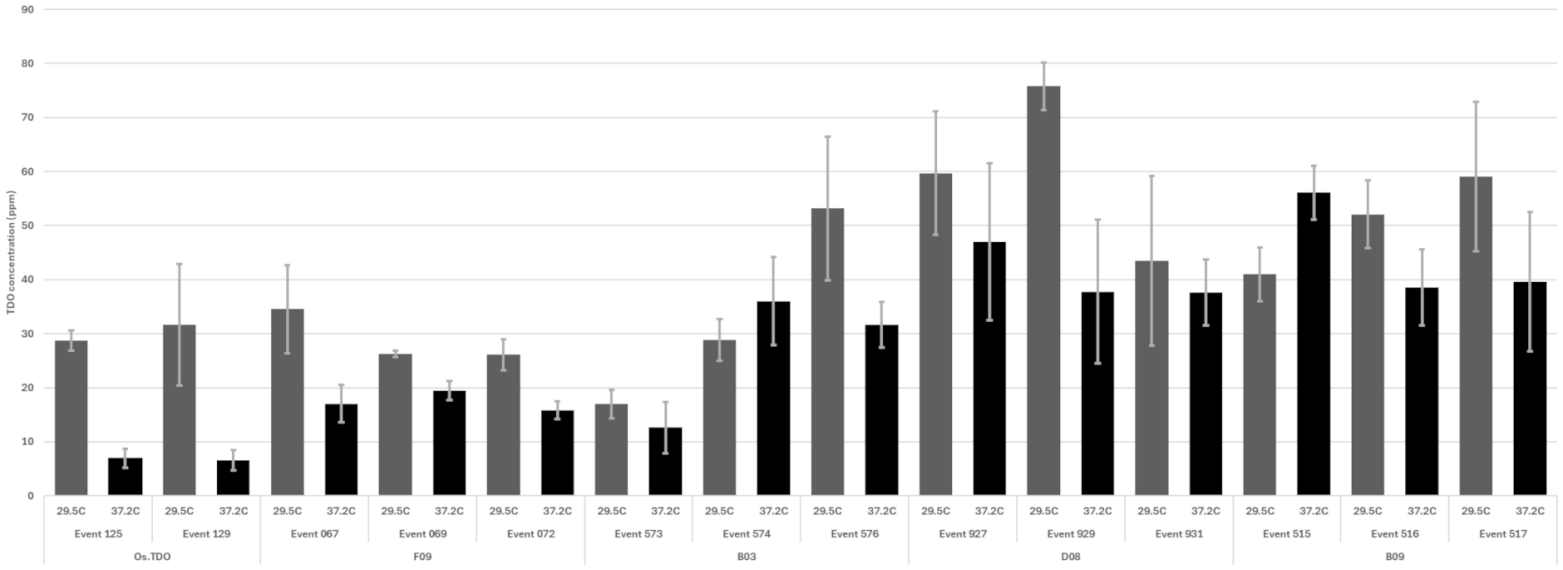
TDO protein accumulation in soybean leaves based on ELISA analysis for round 1 variants. The plants were grown and sampled as described in the Materials and Methods. Error bars are standard error from the mean. TDO, triketone dioxygenase.

**Figure 4:**
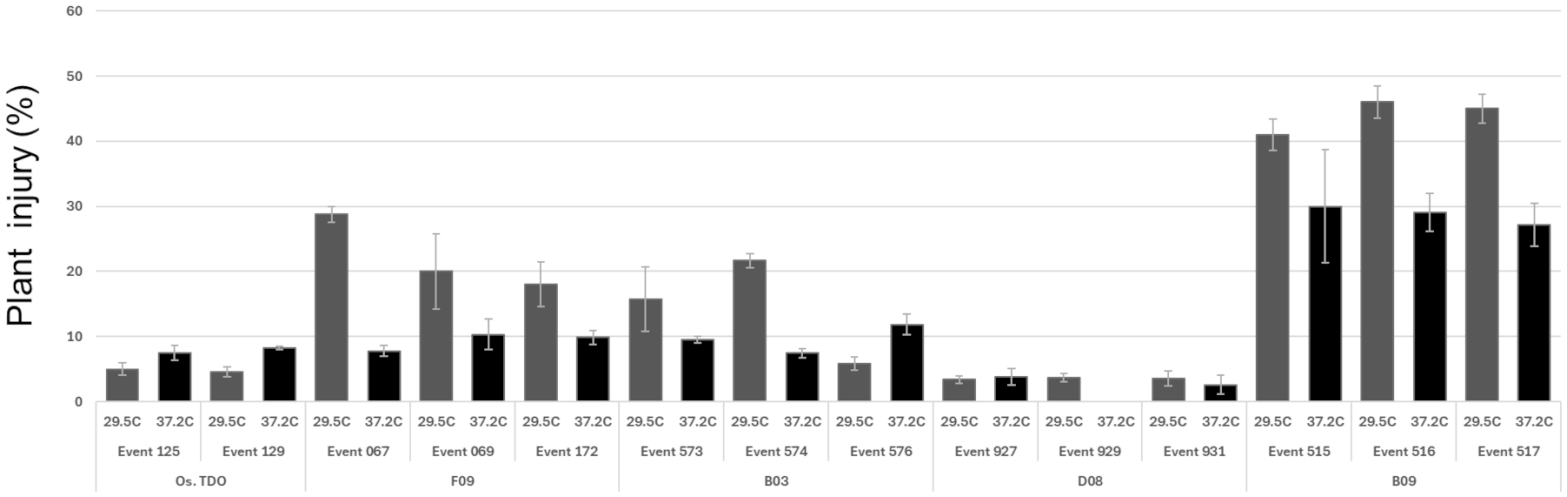
Soybean above-ground injury in the greenhouse and growth chamber for round 1 variants. The plants were grown and tested as described in the Materials and Methods. Error bars are standard error from the mean.

TDO protein accumulation in soybean leaves for the round 1 variants at 27°C and 37.2°C is shown in **Figure 3**. Three of the variants—B03, B09, and D08—had much higher expression than WT TDO at both temperatures. **Figure 4** depicts the crucial plant injury rate for the variants at 27°C and 37.2°C. The lowest injury rate of nearly no visual injury at 37.2°C was observed for D08 event 925, although several other variants and the WT TDO also had injury rates below 10%, indicating very little visual injury. Based on the *in vitro* and *in vivo* data, variant D08 was chosen as the scaffold for a second round of optimization based on restoring the activity lost during round 1.

### 3.4 Kinetic thermal melting point and temperature stability of round 2 variants

The library of round 2 variants was screened as described in the Materials and Methods. The kinetic parameters and T_m_ of the best eight round 2 variants compared to D08 and WT TDO are shown in **Table 3**. Most variants had K_cat_/K_m_ values, which were higher than D08, including variant 205 and variant 225, which had recovered more than half the K_cat_/K_m_ (of D08) lost during the first round from wild-type TDO.

**Table 3:** Kinetic analysis and thermostability of TDO round 2 variants.

Protein	K_cat_ (sec^−1^)	K_m_ (mesotrione) (μM)	K_cat_/K_m_ [M^−1^ sec^−1^] at 25°C(mesotrione)	T_m_ (°C)
Os.TDO	0.12 ± 0.1	4.57	25,764	41
D08	0.07 ± 0.01	12	6,073	47.5
Round 2—0079	0.06 ± 0.00	8.08	7,042	47.5
Round 2—0080	0.07 ± 0.00	8.73	8,114	47.5
Round 2—0087	0.07 ± 0.01	11.09	6,347	47.5
Round 2—0152	0.08 ± 0.03	11.67	6,835	46.5
Round 2—0162	0.08 ± 0.00	18.02	4,498	47.5
Round 2—0205	0.08 ± 0.00	5.23	14,668	47.5
Round 2—0206	0.09 ± 0.0.01	14.69	5,975	45
Round 2—0225	0.08 ± 0.01	6.18	13,580	46.5

All kinetic parameters are the same as those of the second hydroxylation.

TDO, triketone dioxygenase.

**Figure 5:**
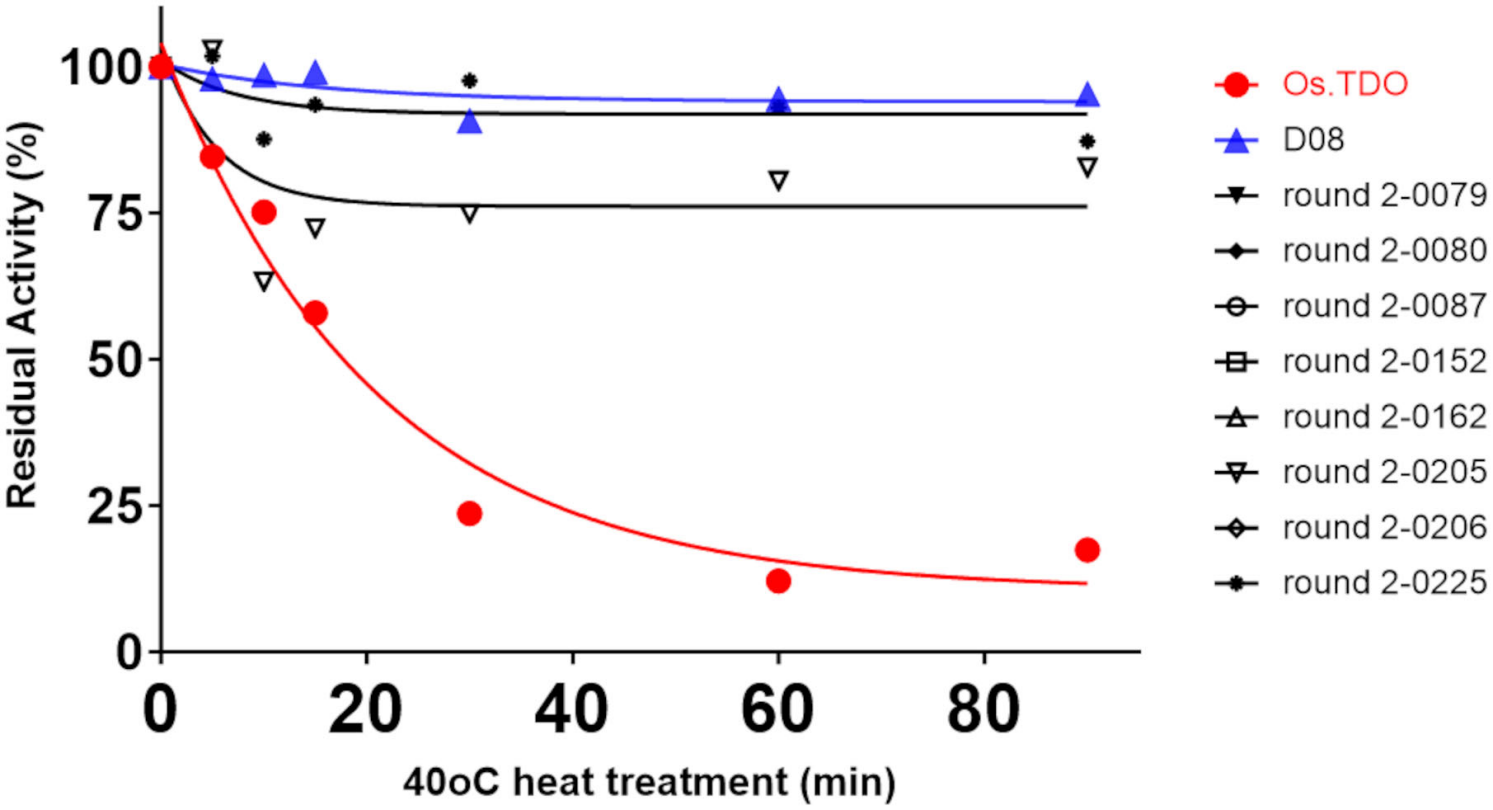
Temperature stability and K_cat_/K_m_ for Os.TDO, D08, and the best round 2 variants. All kinetic parameters are that of the second hydroxylation.

**Figure 5** compares the effect of temperature on the K_cat_/K_m_ values of wild-type, D08, and eight round 2 variants. D08 and all the round 2 variants had K_cat_/K_m_ values considerably lower than those of wild-type TDO; however, the K_cat_/K_m_ values of most of the variants were stable between 25°C and 40°C.

**Figure 6:**
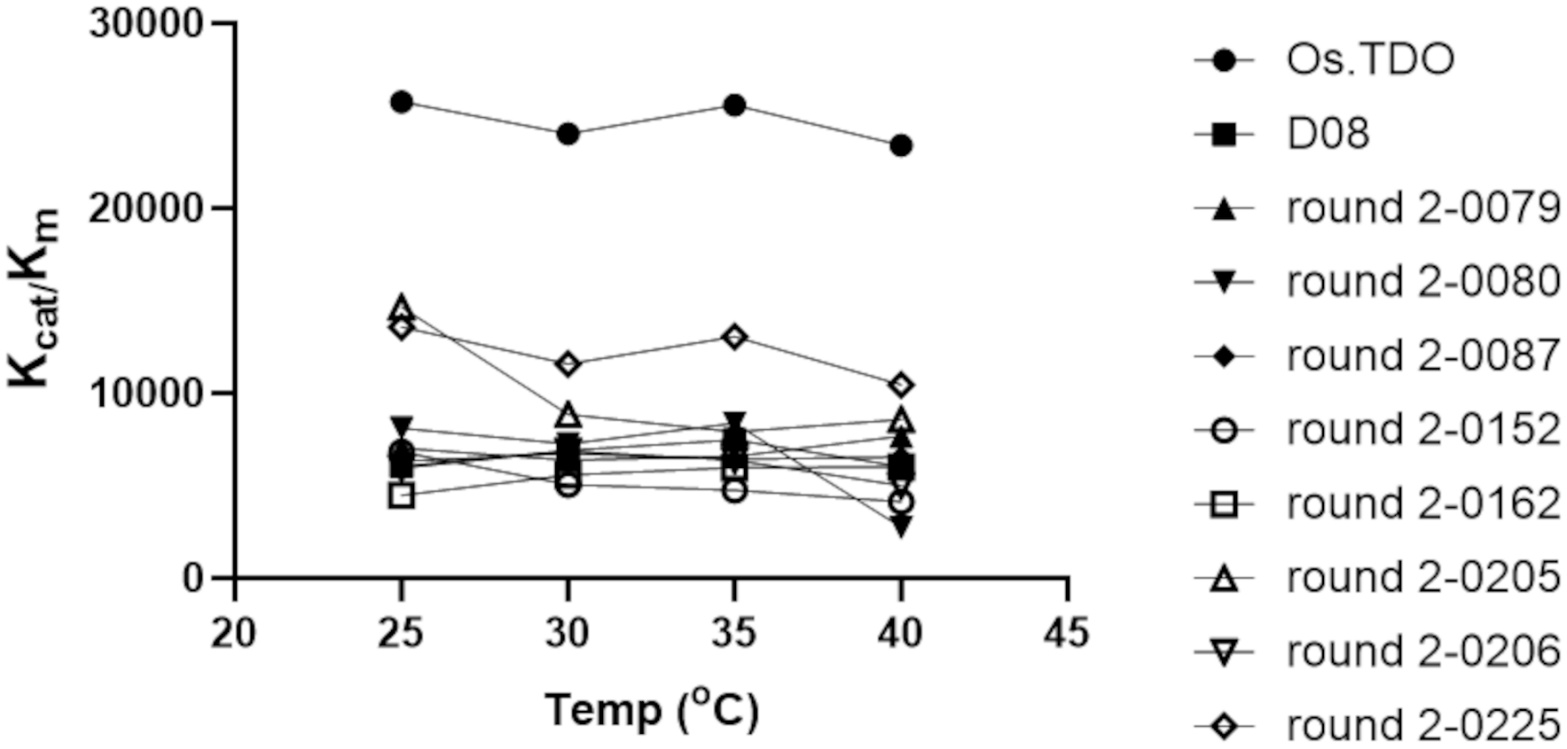
Time course of apoprotein residual activity of the TDO round 2 variants. Os.TDO is emphasized by the red circle, while D08 TDO is emphasized by the blue triangles. Activity was measured for the second hydroxylation.

The stability of wild-type and variant TDO at 40°C is shown in **Figure 6**. Whereas after 30 min, wild-type TDO had lost 75% of its activity, D08 and most of the round 2 variants retained over 90% activity over 90 min.

### 3.5 Growth chamber performance of cotton overexpressing D08 and round 2 TDO variants

Cotton plants overexpressing wild-type D08 and round 2 TDO variants (205 and 225) with the best kinetic parameters were evaluated in the growth chamber at two temperatures, 26.7°C and 37.8°C, and at two separate applied mesotrione concentrations (**Table 2**). Mesotrione treatment led to lower TDO protein expression in all cases at 26.7°C; however, at 37.8°C, the three variants better maintained their TDO protein expression than the wild-type TDO plants. D08 and the round 2 variant 205 displayed no injury regardless of the temperature and mesotrione level in contrast to TDO variant D08-expressing plants, which displayed some injury, and wild-type TDO-expressing plants, which had up to 23% injury at the highest temperature and mesotrione level.

### 3.6 Field performance of D08 and wild-type TDO soybean and cotton plants

Considering the previous results, D08 and wild-type TDO cotton plants were then evaluated in the field for mesotrione injury and agronomic performance (**Table 4**). The D08 variant plants exhibited and then wild-type TDO and wild-type cotton following mesotrione treatment both displayed excellent tolerance with very low visual injury at all the developmental stages that were tested. Broadacre yield measurements were roughly equivalent between D08 and wild-type TDO cotton; however, they both performed as well as wild-type plants in broadacre yield trials.

**Table 4:** Field crop injury and broadacre yield of cotton plants with D08 and WT TDO.

	Mesotrione treatment at 210 g ai/ha			^b^Agronomic performance, total seed cotton, lb/acre	Significance class
	^a^Average crop injury, %				
	PRE_14DAT	V4_7DAT	V8_7DAT		
Os.TDO	3.6	8.3	1.66	3,075	A
Os.TDO_D08	3	7.89	0	3,132	A
WT	30	50		2,978	A

Means followed by the same letters are not significantly different from wild-type control at least significant difference (LSD) at α = 0.05.

DAT, days after treatment; WT TDO, wild-type triketone dioxygenase.

a Crop injury data averaged across four locations and three replications from 2019 US field efficacy trials.

b Mean yield data across eight locations and three replications from 2019 US field agronomic trials.

D08 and wild-type TDO-expressing soybean plants were also evaluated in the field for mesotrione injury and agronomic yield performance. As shown in **Table 5**, field testing confirmed that the D08 variant soybean plants provided excellent tolerance (<4% crop injury) at both the V3 and V8 growth stages but was not significantly different from the wild-type TDO-transgenic soybean plants. Since this testing was conducted in the open field, there was no control over the temperature ranges to which the plants were exposed. The D08 soybean demonstrated good yield performance and did not show a significant difference in agronomic performance from wild-type TDO and non-transgenic soybean in agronomic yield trials.

**Table 5:** Field crop injury and broadacre yield of soybean plants with D08 and WT TDO.

Mesotrione treatment (210 g ai/ha)				Agronomic performance, kg/ha
^a^Averaged crop injury, %			Yield Testing	
PRE_14DAT	V3_7DAT	R1_7 DAT	Yield from treated trial, kg/ha	
Not tested	2.2	2.4	4,317	4,115
Not tested	1	2.2	4,425	Not tested
Not tested	N/A		N/A	4,068

The data shown represent the mean of four locations and three replications from 2017 US field trials.

WT TDO, wild-type triketone dioxygenase.

## 4 Discussion

There are several examples of protein engineering for studying or improving plants. Optimization of *Bacillus thuringiensis* toxin has been a focus of insect control strategies in a variety of ways, including truncation, domain swapping, peptide addition, and amino acid mutation (Deist et al., 2014; Engqvist and Rabe, 2019). EPSPS has been engineered to be less susceptible to glyphosate inhibition by screening libraries of variants generated by DNA shuffling (Tian et al., 2013), error-prone PCR (Mao et al., 2017), or synthetic yeast selection (Reed et al., 2024). In another example, an improved RUBISCO from *Methanococcoides burtonii* has been introduced into plants (Wilson et al., 2016). We have recently developed an optimized enzyme for herbicide tolerance to aryloxyphenoxypropionate (FOP) herbicide and synthetic auxin herbicides, such as 2,4-dichlorophenoxyacetic acid. Optimization of these herbicide tolerance enzyme variants with enhanced enzymatic and temperature stability parameters enabled robust herbicide tolerance from two herbicide families in transgenic maize and soybeans (Larue et al., 2019).

Cotton and soybean are economically important and distantly related dicot crops that are sensitive to mesotrione (8). We have previously reported that TDO can provide commercial-level protection to soybeans (8). Here, we provide further evidence that TDO can provide protection to soybean against mesotrione and provide evidence that TDO can also provide similar protection to cotton.

WT TDO expressed in *Escherichia coli* has a thermo melting point of 39.5°C (**Table 1**; Larue et al., 2019), which led us to question if protein engineering could successfully stabilize the enzyme at higher temperatures. Testing the efficacy of the TDO trait against mesotrione at 29.5°C vs. 37.2°C showed that TDO could provide complete protection against mesotrione at the lower temperature, but the higher temperature reduced the protein accumulation in the plant leaves (**Figure 3**). This suggests that temperature-stabilizing TDO may guard against the observed decrease in enzyme accumulation in heat-treated soybean plants.

In this study, we undertook two rounds of engineering with the purpose of improving the temperature stability and enzyme activity for TDO on mesotrione. In the first round of optimization, we focused on the temperature stability of TDO. However, we quickly found that stabilizing the enzyme was at the expense of catalytic activity, with a significant loss of catalytic efficiency (K_cat_/K_m_) (**Table 1**).

In a soybean growth chamber/greenhouse experiment, round 1 variant D08 provided somewhat better protection to soybean from mesotrione than the wild-type TDO at an artificially maintained higher temperature. Unforeseeably, D08 had approximately 24% of the activity of WT TDO (**Table 3**). We were concerned that the lower activity of D08 could significantly limit its ability to provide herbicide protection in field-grown soybeans exposed to a variety of environmental conditions. Therefore, in the second round of variants, we restored the lost enzymatic activity while maintaining temperature stability. All the round 2 variants had T_m_ values within 1°C of each other and D08. However, we were able to restore the K_cat_/K_m_ from 23.6% of wild-type activity that was observed in D08 to 56.0% of wild-type activity that was observed in the best round 2 variant. In addition, only one of the round 2 finalists decayed significantly more at 40°C than D08.

The best two round 2 variants, D08, and Os.TDO-expressing cotton plants were tested in a growth chamber/greenhouse temperature experiment (**Table 2**), like the soybean test performed on the round 1 variants (**Figures 3**, **4**). In this experiment, D08 TDO protein accumulation was the highest, and the D08 and round 2 variant 225 performed equally well in providing herbicide tolerance. Plants expressing either variant had no visible injury at any of the temperature/mesotrione regimes. This suggests that the temperature stability of these variants, which allowed the enzyme to accumulate to roughly twice the level of the WT TDO in plant leaves at high temperatures, may be able to compensate for the loss of roughly 50% enzyme activity in the stabilized variants. Furthermore, higher temperature stability may be correlated to generally higher stability. Further studies will be needed to substantiate this point.

The D08 variant and Os.TDO were tested in cotton plants in the field. In these experiments, both the D08 variant and the WT Os.TDO showed excellent protection to mesotrione applications and full protection of cotton yield. We can conclude that in cotton, both the D08 variant and the WT Os.TDO variant can provide suitable tolerance to mesotrione under field conditions. Additional testing in soybean also demonstrated excellent tolerance to mesotrione with both variants showing very low visual injury and good yield performance.

## Data Availability

The raw data supporting the conclusions of this article will be made available by the authors, without undue reservation.
